# Seroprevalence of human toxocariasis in Latin America and the Caribbean: a systematic review and meta-analysis

**DOI:** 10.3389/fpubh.2023.1181230

**Published:** 2023-06-27

**Authors:** Juan R. Ulloque-Badaracco, Enrique A. Hernandez-Bustamante, Esteban A. Alarcón-Braga, Miguel Huayta-Cortez, Ximena L. Carballo-Tello, Rosa A. Seminario-Amez, Alejandra Rodríguez-Torres, Donovan Casas-Patiño, Percy Herrera-Añazco, Vicente A. Benites-Zapata

**Affiliations:** ^1^Escuela de Medicina, Universidad Peruana de Ciencias Aplicadas, Lima, Peru; ^2^Sociedad Cientifica de Estudiantes de Medicina de la Universidad Nacional de Trujillo, Trujillo, Peru; ^3^Grupo Peruano de Investigación Epidemiológica, Unidad Para la Generación y Síntesis de Evidencias en Salud, Universidad San Ignacio de Loyola, Lima, Peru; ^4^Universidad Autónoma del Estado de México, CU Amecameca, Mexico; ^5^Red Internacional en Salud Colectiva y Salud Intercultural, Amecameca, Mexico; ^6^Universidad Privada del Norte, Trujillo, Peru; ^7^Red Peruana de Salud Colectiva, Lima, Peru; ^8^Unidad de Investigación Para la Generación y Síntesis de Evidencias en Salud, Vicerrectorado de Investigación, Universidad San Ignacio de Loyola, Lima, Peru

**Keywords:** toxocariasis, zoonoses, seroepidemiologic studies, systematic review, metaanalysis

## Abstract

**Introduction:**

The current study aimed to quantitatively synthesize available evidence regarding the seroprevalence of human toxocariasis in Latin America and the Caribbean.

**Methods:**

A systematic research involving six electronic databases was conducted using a research strategy that combined MeSH terms with free terms. Article selection and information extraction were performed using a double and independent approach. The Newcastle-Ottawa tool was used to assess the risk of bias in the included articles. The meta-analysis used the random-effects approach, with subgroup analysis and sensitivity analysis for risk of bias also being performed.

**Results:**

We included 101 articles with a total of 31,123 participants. The studies were conducted between 1990 and 2022, with Brazil accounting for the largest number of studies (*n* = 37). The overall seroprevalence of human toxocariasis was 31.0% (95% CI: 27.0-35.0%, *I*^2^ = 99%). The prevalence of the main characteristics observed in seropositive patients were as follows: ocular toxocariasis (30.0%), asymptomatic (26.0%), and presence of dogs at home (68.0%). In addition, the seroprevalence was lower in studies including only adults than in those including children or both. In contrast, no differences in seroprevalences were found between studies conducted in the community and hospital.

**Conclusion:**

The overall seroprevalence of human toxocariasis in Latin America and the Caribbean was high. Notably, our findings showed that the seroprevalence was increased among populations who kept a dog at home but was decreased in populations comprising only adults. Our findings can be used to establish epidemiological surveillance strategies for the prevention and early identification of toxocariasis.

## 1. Introduction

Toxocariasis is a zoonosis caused by the larvae of two main species of *Toxocara*, *Toxocara canis* found in dogs and *Toxocara cati* found in cats (1, 2). Although other species, such as *Toxocara leonina* found in foxes, can also cause toxocariasis, *T. canis* has been most associated with human toxocariasis (3). Toxocariasis is a prevalent parasitic disease with a significant socioeconomic impact in symptomatic patients, especially among poor communities, and at the governmental level, with the need for investments in animal deworming campaigns (4, 5). Although infected humans may be asymptomatic, toxocariasis in human always causes extraintestinal pathologies (5). As such, toxocariasis has been associated with various short- and medium-term diseases, such as ocular disease (4), heart disease, stroke, heart failure (6), childhood asthma (7), allergic skin disorders (8), epilepsy (9), or diseases of the urinary system (10), although evidence for this remains inconclusive. Hence, health care systems should prioritize the diagnosis and adequate treatment of this condition.

Toxocariasis is particularly prevalent in the tropics, subtropics, and in low- and middle-income countries where treatment and canine population control is limited (11). However, the prevalence of toxocariasis has varied. Studies estimating the prevalence of specific serum anti-*Toxocara* antibodies found that it was 0.7% in New Zealand, 1.6% in Japan, 2.4% in Denmark, 6.3% in Austria, 7% in Sweden, 14% in the United States, and 31% in Ireland (12). Nevertheless, higher prevalence rates were observed in some ethnic and socioeconomically disadvantaged groups, such as Iran (22%) and Nepal (81%) (12). This suggests that in countries with large socioeconomically disadvantaged groups, better measures are needed to prevent the complications of this disease, including determination of its prevalence based on which future prevention and/or control programs can be implemented (12).

Latin America and the Caribbean are heterogeneous regions with inequities that impact various health indicators (13, 14), including some coexisting conditions that are associated with increased risk of toxocariasis. These include warm, humid regions where eggs survive better in the soil; low levels of education; and areas with a lower human development index and poor environmental sanitation and hygiene (4). Hence, a systematic review found that the prevalence of specific anti-*Toxocara* antibodies was 27.8% in South American countries and 12.8% in North American countries, suggesting potentially frequent infections at the population level (12).

Despite the numerous studies on seroprevalence, to the best of our knowledge, no study has yet synthesized available evidence regarding the seroprevalence of human toxocariasis in Latin American countries according to certain clinical and sociodemographic conditions. Therefore, the present study aimed to synthesize evidence on the seroprevalence of human toxocariasis in Latin America and the Caribbean through a systematic review and meta-analysis.

## 2. Methods

### 2.1. Registration and reporting

A short version of the protocol for this systematic review was uploaded to the International Prospective Register of Systematic Reviews (PROSPERO) [CRD42023389135]. The manuscript follows the Preferred Reporting Items for Systematic Reviews and Meta-Analyzes (PRISMA) statement for reporting results (15).

### 2.2. Search strategy and databases

The search strategy used MeSH, Emtree, and free terms following the Peer Review of Electronic Search Strategies (PRESS) Checklist (16). A search formula was created to retrieve studies assessing the seroprevalence of *T. canis* in humans. Afterwards, it was adopted for all employed databases, without date or language restrictions. Preprint databases and reference lists of the included studies were also manually searched. A systematic search of the following databases was performed simultaneously on December 23, 2022: PubMed, Scopus, Embase, LILACS, Scielo, and Web of Science. The complete search strategy is detailed in Supplementary Table S1 attached as Supplementary material.

### 2.3. Study selection and data extraction

All phases of study selection were independently performed by two authors (E.A.A-B and E.A.H-B). The eligibility criteria were (1) cross-sectional studies assessing the (2) seroprevalence of *T. canis* in humans. We excluded narrative reviews, scoping reviews, systematic reviews, and conference abstracts. Duplicates were removed using EndNote 20.1 ©. The remaining studies were then exported to Rayyan QCRI © for screening according to titles and abstracts (17). After identifying potential studies for inclusion, the authors independently assessed the full text of each one. Any conflict or discrepancy in any phase of the study selection process was resolved via consensus. Data extraction was independently performed by four authors (J.R.U-B, X.L.C-T, R.A.S-A and M.A.H-C) using a standardized data extraction sheet created using Microsoft Excel ©. The following pieces of information were extracted: author names, publication date, country, type population, sample size, survey modality, and seroprevalence of human toxocariasis in Latin America and the Caribbean.

### 2.4. Risk of bias and publication bias

Quality assessment was performed independently by four authors (J.R.U-B, X.L.C-T, R.A.S-A and M.A.H-C). We used the Newcastle-Ottawa Scale (NOS) for cohort/case–control studies and the adapted version of the Newcastle-Ottawa Scale for cross-sectional studies (NOS-CS) (18). Studies with a score of ≥7 stars were considered to have a low risk of bias (high quality), whereas those with a score of <7 stars were considered to have a high risk of bias (low quality). Publication bias was not assessed given that it is not recommended for proportional meta-analyzes considering the lack of evidence that proportions are adjusted correctly (19, 20).

### 2.5. Data synthesis

Statistical analysis was performed using STATA 17.0 ©. We conducted a pooled analysis of the seroprevalences of human infections according to *T. canis*. The 95% confidence intervals (CI) for the proportions reported in each study were calculated using the Clopper–Pearson method. The Freeman–Tukey double arcsine transformation was used as the variance stabilizer. A random-effects model (Dersimonian and Laird) was created for quantitative analysis. Between-study heterogeneity was assessed using the Cochran’s Q test and I^2^ statistic. Values ≥60% indicated high heterogeneity for the I^2^ statistic, whereas *p* values <0.1 indicated heterogeneity in Cochran’s Q test. In addition, we conducted a subgroup analysis according to continent and population groups and a sensitivity analysis excluding studies with high risk of bias.

## 3. Results

### 3.1. Search results

**Figure 1:**
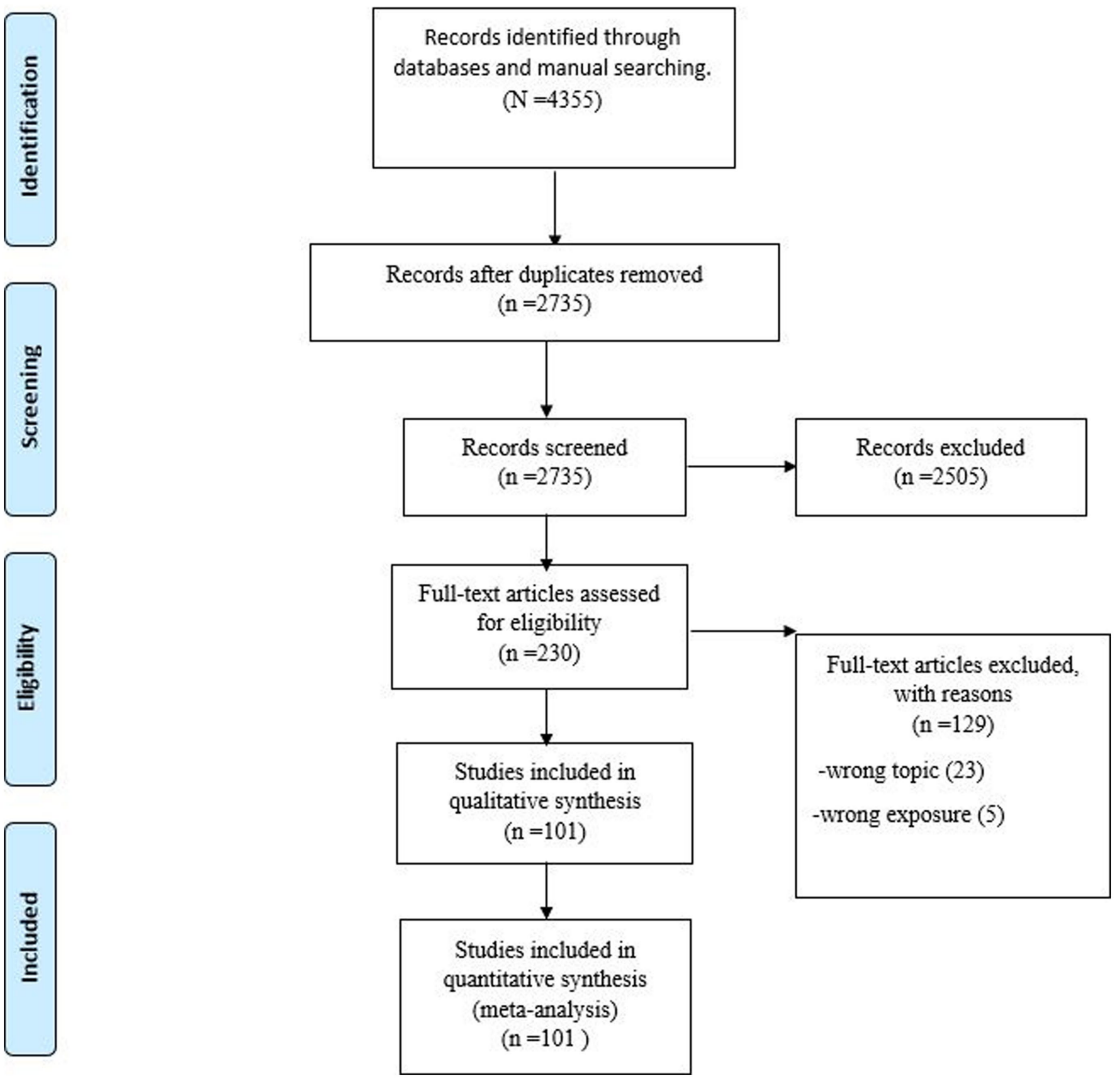
PRISMA flow diagram.

The systematic search identified a total of 4,355 studies. After removing 1,620 duplicate studies, 2,735 studies remained for further screening. Subsequently, screening according to abstracts and titles was performed, after which 230 studies remained. Finally, the full texts of the remaining studies were evaluated, and 101 studies were found to satisfy all eligibility criteria (21–121). This selection process is summarized in the PRISMA flow diagram (Figure 1).

### 3.2. Study characteristics

A total of 101 studies with 31,123 participants were included. The studies were conducted between 1990 and 2022 and were developed in the following countries: Brazil (35 studies), Argentina (13 studies), Venezuela (10 studies), Mexico (10 studies), Peru (11 studies), Colombia (5 studies), Bolivia (3 studies), Cuba (3 studies), Chile (2 studies), Ecuador (2 studies), Puerto Rico (1 study), Trinidad and Tobago (1 study), Saint Lucia (1 study), Paraguay (1 study), Honduras (1 study), Jamaica (1 study), and multiple Caribbean countries (1 study). In all studies, seroprevalence was assessed using ELISA method (Table 1).

**Table 1:** Characteristics of the included studies.

Author	Year	Country	Type of population	Sample collection site	Participants (male/female)	Number of seropositive participants	Seroprevalence of *Toxocara* infection	Ocular toxocariasis in seropositive participants	Seropositive participants with dogs at home	Asymptomatic seropositive participants
Acero M et al.	2001	Colombia	Children	Community	193 (114/79)	14	7.30%	NR	8	NR
Agudelo C et al.	1990	Colombia	Children and Adult	Community	202 (NR/NR)	95	47.50%	NR	NR	NR
Aguiar-Santos AM et al.	2004	Brazil	Children	Hospital	386 (269/117)	152	39.40%	NR	NR	NR
Alderete J et al.	2003	Brazil	Children	Community	399 (196/203)	155	38.80%	NR	NR	NR
Alonso JM et al.	2004	Argentina	Children and adult	Hospital	355 (30/325)	138	38.00%	NR	NR	NR
Alvarado-Esquivel C et al. (A)	2013	Mexico	Adults	Hospital	90 (34/56)	12	13%	4	1	NR
Alvarado-Esquivel C et al. (B)	2014	Mexico	Children and Adult	Community	126 (53/73)	33	26.20%	NR	NR	NR
Alvarado-Esquivel C et al. (C)	2014	Mexico	Children and Adult	Community	336 (306/30)	6	1.80%	NR	NR	NR
Anaruma Filho F et al.	2002	Brazil	Children and Adult	Community	138 (87/51)	33	23.90%	NR	9	NR
Araujo Z et al.	2015	Venezuela	Children and Adult	Community	50 (21/29)	5	10%	NR	NR	NR
Archelli S et al. (A)	2014	Argentina	Children	Community	120 (NR/NR)	46	38.33%	NR	NR	NR
Archelli S et al. (B)	2018	Argentina	Children and Adult	Community	98 (NR)	40	40.80%	NR	NR	NR
Baboolal S et al.	2002	Trinidad and Tobago	Children	Community	1,009 (498/511)	629	62.33%	NR	NR	NR
Berrocal J et al.	1980	Puerto Rico	Children and Adult	Hospital	671 (NR/NR)	44	6.50%	NR	NR	NR
Bojanich MV et al.	2008	Argentina	Children	Community	273 (144/129)	168	61.54%	NR	153	NR
Cabral M et al.	2022	Brazil	Children	Community	412 (NR/NR)	234	56.80%	NR	179	NR
Campos-Junior D et al.	2003	Brazil	Children	Hospital	602 (NR/NR)	75	12.45%	NR	NR	NR
Cancrini G et al.	1998	Bolivia	Adults	Community	216 (NR/NR)	73	34%	NR	NR	NR
Cermeño J et al.	2016	Venezuela	Children	Community	146 (73/73)	43	32.20%	1	43	29
Chieffi P et al.	1990	Brazil	Children and Adult	Community	2025 (1,219 /806)	70	3.46%	NR	NR	NR
Chiodo P et al.	2006	Argentina	Children and Adult	Community	100 (46/54)	23	23%	NR	23	NR
Coelho LM et al.	2004	Brazil	Children	Community	180 (92/88)	69	38.33%	NR	53	NR
Colli C et al.	2010	Brazil	Children	Community	376 (NR/NR)	194	51.60%	NR	NR	NR
Cook J et al.	2016	Jamaica	Children and Adult	Community	1,447 (738/709)	304	21%	NR	NR	NR
Correa C et al.	2009	Brazil	Children	Community	100 (NR/NR)	28	28%	NR	NR	28/28
Damian M et al.	2007	Brazil	Children and Adult	Community	100 (NR/NR)	52	52%	NR	NR	NR
Dattoli V et al.	2011	Brazil	Adults	Community	306 (108/198)	124	46.30%	NR	NR	NR
De Abreu A et al.	2011	Venezuela	Children	Community	215 (112/103)	74	34.40%	NR	NR	NR
Devera R et al.	2015	Venezuela	Children	Community	547 (223/3244)	269	58.90%	NR	206	NR
Díaz-Suárez O et al.	2010	Venezuela	Children and Adult	Community	83 (42/41)	18	21.68%	NR	NR	NR
Espinoza Y et al. (B)	2008	Peru	Children	Community	182 (NR/NR)	59	32.40%	36	58	1
Espinoza Y et al. (A)	2010	Peru	Children and Adult	Community	303 (128/175)	62	20.46%	11	NR	NR
Espinoza Y et al. (C)	2003	Peru	Children and Adult	Hospital	553 (NR/NR)	129	23.32%	NR	NR	NR
Figuereido S et al.	2005	Brazil	Children	Hospital	208 (NR/NR)	114	54.80%	NR	NR	NR
Fillaux J et al.	2007	Argentina	Children and Adult	Hospital	114 (45/69)	36	31.57%	NR	14	NR
García-Pedrique ME et al.	2004	Venezuela	Children	Community	72 (45/27)	7	9.72%	NR	NR	NR
Gétaz-Schaller L et al.	2007	Peru	Children	Hospital	150 (72/78)	24	16%	NR	15	NR
Guilherme E et al.	2013	Brazil	Children	Hospital	167 (NR/NR)	7	4.20%	NR	5	0
Guo F et al.	2016	Multiple Caribbean countries	Adults	Hospital	435 (0/435)	63	14.50%	NR	NR	NR
Heredia R et al.	2014	Mexico	Adults	Community	200 (NR/NR)	20	10%	NR	15	NR
Hernández S et al.	2020	Honduras	Children and Adult	Community	88 (43/45)	80	90.90%	NR	NR	NR
Kanobana K et al.	2013	Cuba	Children	Hospital	958 (504/454)	384	40.10%	NR	NR	NR
Lescano S et al.	1998	Peru	Children and Adult	Community	1,023 (740/283)	75	7.33%	NR	NR	NR
Lima-Coêlho R et al.	2005	Brazil	Children	Community	215 (NR/NR)	26	12.09%	NR	NR	NR
Lopez MA et al.	2010	Argentina	Children	Hospital	100 (NR/NR)	55	55%	NR	NR	NR
Lozano-Beltrán D et al.	2011	Bolivia	Children	Hospital	82 (NR/NR)	5	6.50%	NR	5	NR
Lynch NR et al. (A)	1988	Venezuela	Children	Hospital	476 (NR/NR)	56	11.7%	NR	37	NR
Lynch NR et al. (B)	1993	Venezuela	Children	Hospital	368 (NR/NR)	74	20.10%	NR	NR	NR
Manini M et al.	2012	Brazil	Children	Community	90 (NR/NR)	16	17.80%	NR	NR	NR
Marchioro A et al.	2015	Brazil	Children	Community	544 (299/245)	136	25%	NR	NR	NR
Martín U et al. (A)	2014	Argentina	Children	Community	857 (NR/NR)	335	59.70%	NR	NR	NR
Martín U et al. (B)	2008	Argentina	Children	Community	100 (62/38)	59	59%	5	NR	NR
Martínez M et al.	2018	Venezuela	Children	Hospital	259 (148/111)	37	14.30%	5	25	NR
Mattia S et al.	2011	Brazil	Children	Community	353 (NR/NR)	130	36.80%	NR	78	NR
Meza D et al.	2010	Colombia	Children	Community	133 (57–76)	56	42.10%	1	38	NR
Minvielle M et al.	2000	Argentina	Adults	Hospital	180 (47/133)	19	10.60%	NR	NR	NR
Miranda-Choque E et al.	2014	Peru	Children	Hospital	242 (92/150)	148	61.20%	16	NR	NR
Montalvo A et al.	1994	Cuba	Children	Community	152 (NR/NR)	8	5.20%	NR	NR	NR
Morocoima A et al.	2021	Venezuela	Children	Hospital	118 (50/68)	22	18.60%	14	NR	NR
Muñoz-Guzmán M et al.	2010	Mexico	Children	Hospital	437 (NR)	118	27%	NR	NR	NR
Muradian V et al.	2005	Brazil	Children	Community	338 (NR/NR)	89	26–9%	NR	NR	NR
Nava-Cortés N et al.	2015	Mexico	Children	Community	183 (97/86)	22	12.02%	NR	8	NR
Negri E et al.	2013	Brazil	Adults	Community	253 (148/105)	22	8.70%	NR	14	NR
Nicoletti A et al.	2002	Bolivia	Children and Adult	Community	346 (NR/NR)	56	24.80%	NR	NR	NR
Oliart-Guzmán H et al.	2014	Brazil	Children	Community	539 (NR/NR)	106	19.66%	NR	NR	NR
Orlando-Indacochea N et al.	2018	Ecuador	Children	Community	50 (NR)	18	36%	NR	NR	NR
Ortega-Pacheco A et al.	2015	Mexico	Adults	Community	84 (8/76)	19	22.60%	16	NR	NR
Oviedo-Vera A et al.	2021	Ecuador	Children	Hospital	290 (NR)	200	69%	NR	NR	NR
Paludo M et al.	2007	Brazil	Children	Hospital	130 (NR/NR)	36	28.80%	NR	78	NR
Paranhos-Fragoso et al.	2011	Brazil	Children	Hospital	391(NR/NR)	202	51.60%	NR	78	NR
Pereira L et al.	2016	Brazil	Adults	Hospital	311 (0/311)	23	7.20%	NR	NR	NR
Prestes-Carneiro L et al. (A)	2008	Brazil	Children	Community	79 (NR/NR)	17	21.50%	NR	NR	NR
Prestes-Carneiro L et al. (B)	2009	Brazil	Children and Adult	Community	182 (64/118)	25	13.73%	8	20	4
Prestes-Carneiro L et al. (C)	2013	Brazil	Children and Adult	Community	194 (101/93)	102	52.60%	NR	67	NR
Pulcha-Ugarte R et al.	2021	Peru	Children	Community	61 (33/28)	22	36.10%	NR	7	NR
Radman NE et al.	2000	Argentina	Children and Adult	Hospital	156 (NR/NR)	61	39.10%	4	NR	10
Ramírez-Bustamante C et al.	2010	Peru	Children and Adult	Hospital	41 (21/20)	31	75.60%	31	NR	NR
Ribeiro L et al.	2012	Brazil	Children	Community	1,445 (NR/NR)	540	47%	NR	NR	NR
Rivarola M et al.	2009	Paraguay	Children	Community	68 (NR)	53	78%	NR	NR	NR
Rodriguez C et al.	2006	Brazil	Children	Hospital	242 (NR/NR)	21	8.70%	NR	NR	NR
Roldan W et al. (A)	2008	Peru	Children	Community	200 (96/104)	62	31%	13	43	NR
Roldan W et al. (B)	2009	Peru	Adults	Community	256 (NR/NR)	115	44.92%	16	109	27
Roldan W et al. (C)	2010	Peru	Children and Adult	Community	300 (112/188)	107	35.66%	39	89	5
Romero R et al.	2017	Colombia	Children	Community	165 (77/88)	30	18.20%	NR	NR	NR
Romero-Nuñez C et al.	2013	Mexico	Children	Community	108 (52/56)	24	22.22%	NR	18	NR
Rubinsky-Elefant G et al.	2008	Brazil	Children and Adult	Community	403 (NR/NR)	108	26.80%	NR	31	NR
Santarem V et al.	2011	Brazil	Children	Community	252 (NR/NR)	28	11.10%	NR	18	NR
Santos P et al.	2015	Brazil	Children and Adult	Community	280 (0/280)	18	6.40%	NR	16	NR
Sariego I et al.	2012	Cuba	Children	Community	1,011 (525/486)	392	38.80%	NR	NR	NR
Schoenardie E et al.	2013	Mexico	Children	Hospital	427 (NR/NR)	216	50.58%	NR	NR	NR
Silva-Cadore P et al.	2016	Brazil	Children	Hospital	208 (113/95)	24	11.53%	NR	NR	NR
Souza RF et al.	2011	Brazil	Children and Adult	Community	338 (NR/NR)	201	59.46%	NR	120	NR
Taranto NJ et al. (A)	2000	Argentina	Children	Community	98 (NR/NR)	20	20.40%	NR	NR	NR
Taranto NJ et al. (B)	2003	Argentina	Children and Adult	Community	154 (NR/NR)	34	22.10%	NR	NR	NR
Thompson DE et al.	1986	Saint Lucía	Children	Hospital	203 (NR/NR)	170	84%	NR	NR	NR
Tinoco-Gracias L et al.	2008	Mexico	Children	Hospital	288 (153/135)	25	8.68%	NR	10	NR
Triviño X et al.	1999	Chile	Children	Hospital	24 (NR)	8	33.30%	1	8	0
Urbano-Ferreira M et al.	2007	Brazil	Children	Community	606 (NR/NR)	133	21.50%	NR	NR	NR
Vargas C et al.	2016	Chile	Children and Adult	Hospital	355 (NR/NR)	90	25.40%	NR	80	NR
Virginia P et al.	1991	Brazil	Children	Hospital	54 (NR/NR)	21	40%	NR	NR	NR
Waindok P et al.	2021	Colombia	Children and Adult	Hospital	483 (232/247)	383	79.30%	NR	NR	NR

NR, not reported.

During the quality assessment of the studies using NOS and NOS-CS, 5 studies were found to have a high risk of bias, whereas the remaining 96 were found to have a low risk of bias (Supplementary Table S2).

### 3.3. Seroprevalence of human toxocariasis

Some meta-analyzes results are summarized in Table 2 given the large number of studies involved, which diminish the quality of the images. All 101 studies were included in the meta-analysis. Notably, the seroprevalence of human toxocariasis in Latin America and the Caribbean was 31.0% (95% CI: 27.0–35.0%) with high heterogeneity across all studies (I^2^ = 99%). Subgroup analysis according to country showed no decrease in heterogeneity in any of the countries: Brazil (28%), Argentina (37%), Venezuela (22%), Mexico (19%), Peru (34%), Colombia (39%), Bolivia (19%), Cuba (28%), Chile (26%), and Ecuador (64%). Analysis according to age group revealed no decrease in heterogeneity in any of the groups: Children and adults (31%), adults (20%), and children (32%). Subgroup analysis according to sample collection site found no decrease in heterogeneity in any of the groups: Community (30%) and Hospital (31%). Finally, sensitivity analysis revealed no evidence of a decrease in heterogeneity with a seroprevalence of 30.0% (95% CI: 26.0–34.0%, I^2^ = 99.03).

**Table 2:** Results of seroprevalence meta-analyzes.

	Number of studies	Pooled seroprevalence (%)	95% CI	*n*	*I*^2^	*p*
Overall seroseroprevalence	101	31.0%	27.0–35.0%	31,123	99%	<0.001
Central America and Caribbean Islands	19	–	–	8,253		
Mexico	10	19.0%	10.0–29.0%	2,279	98.23%	<0.001
Cuba	3	28.0%	7.0–49%	2,121	99.23%	<0.001
Puerto Rico	1	6.0%	4.0–9.0%	671	–	<0.001
Trinidad and Tobago	1	62.0%	59.0–65.0%	1,009	–	<0.001
Saint Lucia	1	83.0%	78.0–88.0%	203		<0.001
Honduras	1	90.0%	83.0–86.0%	88	–	<0.001
Jamaica	1	21.0%	18.0–23.0%	1,447	–	<0.001
Multiple Caribbean Countries	1	14.0%	11.0–18.0%	435	–	<0.001
South America	82	–	–	22,870		
Brazil	35	28.0%	22.0%–34.0%	12,508	98.89%	<0.001
Argentina	13	37.0%	28.0–45.0%	2,409	95.76%	<0.001
Venezuela	10	22.0%	13.0–31.0%	2035	96.57%	<0.001
Peru	11	34.0%	23.0%–46.0%	3,311	98.34%	<0.001
Colombia	5	39.0%	6.0–71.0%	1,176	99.51%	<0.001
Bolivia	3	19.0%	5.0–32.0%	644	95.49%	<0.001
Chile	2	26.0%	21.0–30.0%	379	-	<0.001
Ecuador	2	64.0%	59.0–69.0%	340	-	<0.001
Paraguay	1	78.0%	66.0–87.0%	68	-	<0.001
Type of population						
Children	61	32.0%	27.0–37.0%	17,786	98.48%	<0.001
Adults	10	20.0%	13.0–28.0%	2,293	96.54%	<0.001
Childrens and adults	30	31.0%	25.0–38.0%	1,104	99.26%	<0.001
Sample collection site						
Community	66	30.0%	26.0–35.0%	10,439	98.98%	<0.001
Hospital	36	31.0%	24.0–38.0%	5,144	99.02%	<0.001
Sensitivity analysis	96	30.0%	26.0–34%	30,436	99.03%	<0.001

### 3.4. Characteristics of patients seropositive for human toxocariasis

**Figure 2:**
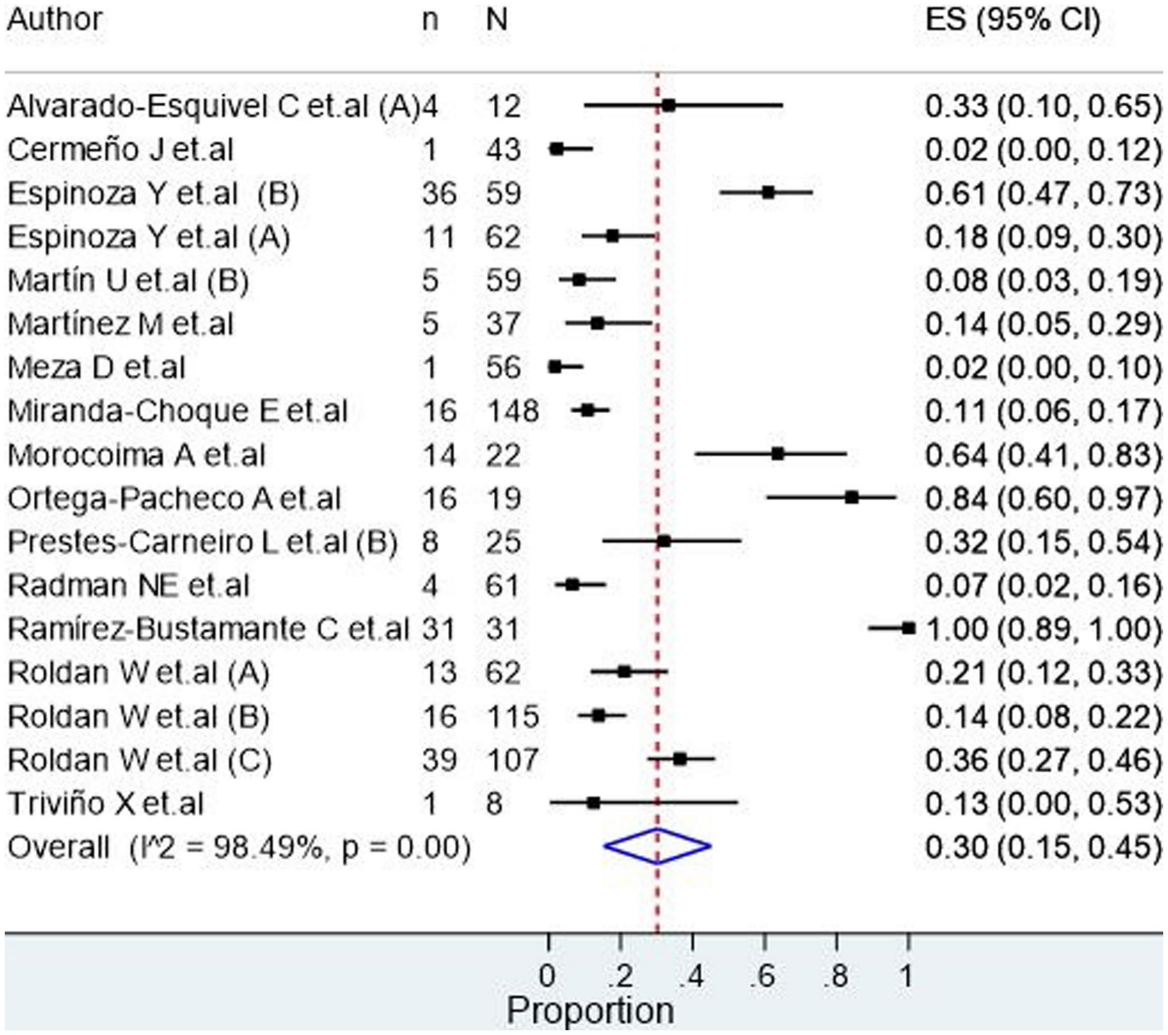
Seroprevalence of ocular toxocariasis in seropositive participants.

**Figure 3:**
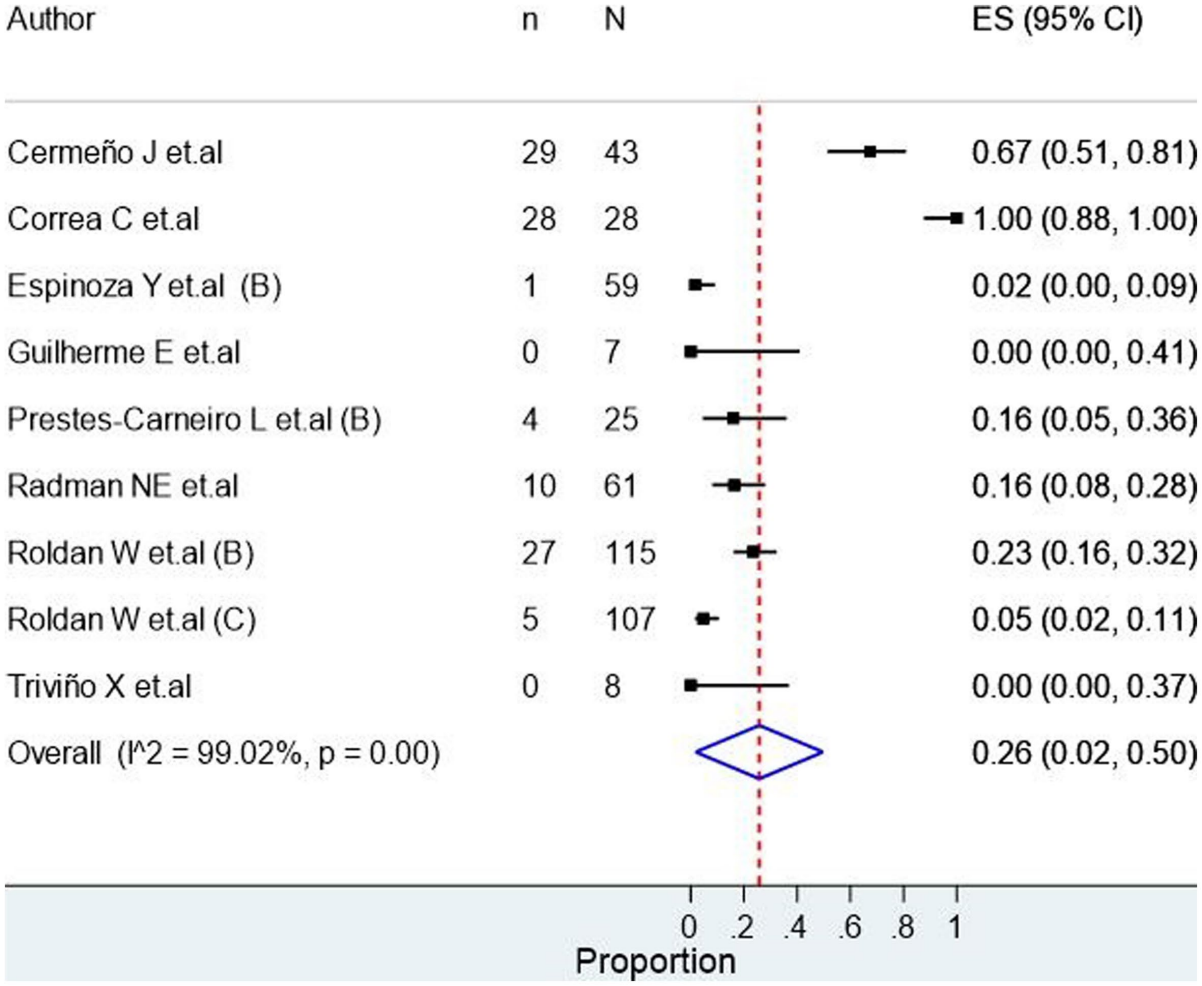
Seroprevalence of asymptomatic seropositive participants.

**Figure 4:**
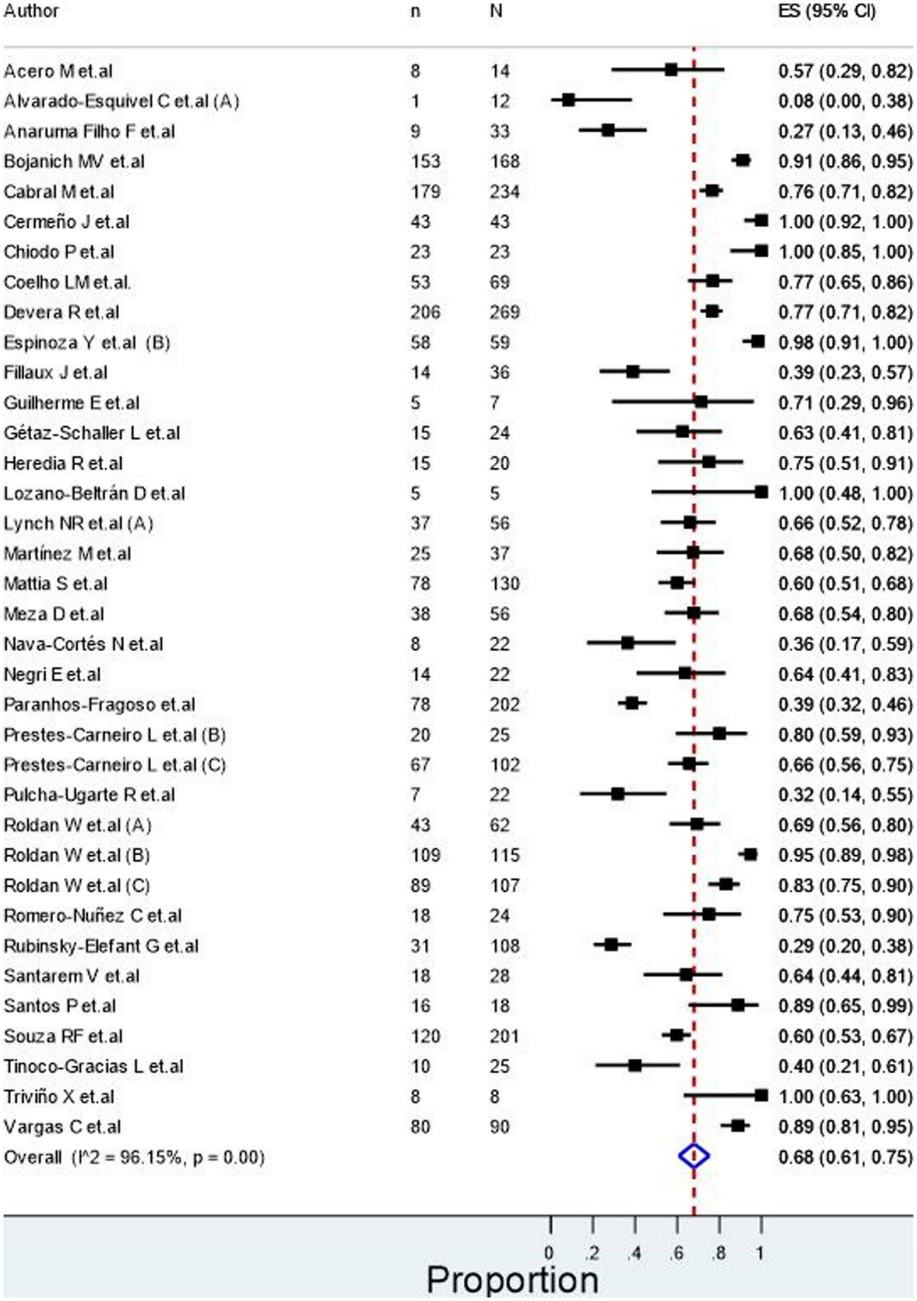
Seroprevalence of seropositive participants with dogs in home.

Ocular toxocariasis in patients with human toxocariasis was evaluated in 17 studies (Figure 2), revealing a prevalence of 30.0% (95% CI: 15.0–45.0%) with a high heterogeneity among studies (I^2^ = 98.49%). Meanwhile, the number of asymptomatic patients with human toxocariasis was evaluated in 9 studies (Figure 3), revealing a prevalence of 26.0% (95% CI: 2.0–50.0%) with a high heterogeneity among studies (I^2^ = 99.02%). Finally, the seroprevalence of toxocariasis in patients with dogs at home was evaluated in 36 studies, revealing a prevalence 68.0% (95% CI: 61.0–75.0%; Figure 4) with a high heterogeneity among studies (I^2^ = 96.15%).

## 4. Discussion

The main findings of the current study show that 3 out of 10 participants were seropositive for *T. canis*, with the same findings obtained at the community and hospital levels. A comparison between children and adults showed that 3 out of 10 children and 2 out of 10 adults were seropositive for *T. canis*. Meanwhile, two-thirds of patients living with dogs in homes were seropositive. Variations in seroprevalence had been noted among countries, with Honduras and Puerto Rico showing an increased and decreased seroprevalence, respectively. One-fourth of the seropositive patients were asymptomatic and one-third of them had ocular involvement.

The worldwide prevalence of *Toxocara* in humans is influenced, at the population level, by environmental, geographic, cultural, and socioeconomic factors and, at the individual level, by susceptibility to infection, which is consequently influenced by immunity, co-infection, genetics, age, nutrition, or the sex of the host (11). These factors, along with population growth, global migration, and rural–urban migration, have promoted increasingly close human–host interactions, making toxocariasis a dynamic public health problem (11). However, some systematic reviews have attempted to determine its seroprevalence.

A systematic review that calculated the overall prevalence of serum anti-*Toxocara* antibodies in humans found that one-fifth of the world’s population is seropositive for *Toxocara*, with Africa showing increased seropositive rates with a mean of 37.7% and the Eastern Mediterranean region showing decreased rates with a mean of 8.2%. Evidencing in South American countries, specifically in Latin America and the Caribbean, showed an average seroprevalence of 27.8% (range 23.1–32.7%) and was 12.8% in North American countries (range 10.0–15.8%) (122). These results show the heterogeneity of prevalence in the countries within the region, similar to that observed herein and in various systematic reviews involving other regions globally.

In fact, a systematic review that determined the burden of toxocariasis in North America found that prevalence estimates ranged from 0.6% in a Canadian Inuit community to 30.8% in Mexican children with asthma (123). In Africa, another systematic review found that, despite underdiagnosis, human exposure to *Toxocara* is widespread in various geographical areas and different populations in Africa and occurs in virtually all climatic zones, with seroprevalences greater than 80% in some populations such as in Makoko, Nigeria, or La Réunion (124). Finally, the seroprevalence among the general European population was 6.2%, although no significant differences were observed among the combined prevalence rates of the European subregions (125).

These differences may be due to the varying proportion of risk factors in the studies, which also vary among the regions studied and, in some cases, are common in Latin American and Caribbean countries. Globally, areas with a low human development index and income levels, those located within the low-latitude regions, those having high ambient temperature, and those with increased precipitation have been associated with a high prevalence of serum anti-*Toxocara* antibodies (122). Other risk factors include male sex; living in rural areas; young age; close contact with dogs, cats, or soil; consumption of raw meat; and consumption of untreated drinking water (122). In North America, commonly cited risk factors include African American race, poverty, male sex, pet ownership, or environmental contamination by animal feces (123). Additionally, apart from the differences in serodiagnostic methods used, other systematic reviews likely included other types of *Toxocara*, unlike our research, which only evaluated the seroprevalence of *T. canis*, precluding direct comparisons between our results (5). Likewise, a limitation of *T. canis* antigen tests, which is their cross-reactivity with other helminths such as *Ascaris lumbricoides*, needs to be considered, particularly in endemic areas (5) given the potential for overestimating the seroprevalence of *T. canis* in Latin America and the Caribbean (126).

Our study, which evaluated not only overall seroprevalence but also differences according to site of evaluation and age group, found that around 3 out of 10 seropositive patients were asymptomatic. In most patients, *Toxocara* infections were not severe, and several people, especially adults infected with a small number of larvae, may remain asymptomatic or have mild or nonspecific symptoms that go undiagnosed, with more severe cases being rare (4). In this sense, the similarity in seroprevalence at the hospital and community levels could be attributed the low disease severity in majority of the cases. Similarly, although the percentage of asymptomatic patients in our study is important, we emphasize that positive results in serological test for antibodies against *Toxocara*, which have been available, are not necessarily correlated with any clinical symptoms and cannot differentiate between current active disease and past infection (4).

Another significant finding of our study was that 3 out of 10 seropositive patients had ocular involvement. Ocular involvement in toxocariasis occurs when *Toxocara* larvae migrate to the eyes, promoting symptoms and signs that include vision loss, ocular inflammation, or retinal damage, usually in a single eye (127). As of 2018, a total of 823 cases of ocular toxocariasis had been reported, including 282 cases in Europe, 317 cases in Asia, five cases in Australia, 218 cases in Latin America, and 1 documented case in Tunisia (4). The highest number of ocular toxocariasis cases had been reported in Japan, Korea, France, Brazil, and the US (4). Our results showed that up to 133 Peruvian patients alone were seropositive, which warrants special follow-up by the Peruvian health authorities. Ocular involvement is more common among children aged 5 to 10 years (128–130), which could explain the higher prevalence of toxocariasis among children. However, studies in Europe show otherwise such that the pooled seroprevalence was higher among people over 50 years of age than among younger people (18). A systematic review found that the pooled estimate of the worldwide prevalence of *T. canis* in the pediatric population was 30%, with Asian populations showing higher rates than the European, American, and African populations (131). One study in America showed a seroprevalence of 31%; however, given that this study included North American countries and both *T. canis* and *T. cati*, our results cannot be compared (131).

Our findings showed that the seroprevalence was higher in patients who had a dog in the household. This is consistent with the results of a systematic review in the Americas, Middle East, and Western Pacific Region, which found a statistically significant association between seropositivity for *Toxocara* and contact with pets, although only in younger people with both dogs and cats (132). Toxocariasis has a fecal–oral route of transmission where domestic dogs accounted for 39% of the total egg production, followed by feral cats, domestic cats, and foxes (4). However, in some urban areas, egg production may be dominated by feral cats (4). Therefore, some health care systems include mass deworming of dogs and cats as a strategy for disease control. However, attention has been drawn to harmful effects of parasiticides on a wide range of invertebrates, which could be very detrimental to wildlife and ecosystems and could impact public health. Another important strategy could be the use of molecular tests (polymerase chain reaction) for the identification of various types of *Toxocara* eggs in parks and recreational areas for better monitoring and control.

To avoid these problems, one study in the United Kingdom suggest promoting regular health checks and providing information on all approaches to prevent animals from contracting parasites, explaining the symptoms, informing owners regarding the risks associated with infections, and reminding them to always collect and dispose of feces responsibly (4). Likewise, the same study suggested providing an explanation to patients and animal owners regarding the potential risks of parasiticides to animals, humans, and the environment. Finally, the mentioned study suggests adopting a risk-based approach to prescribing in which parasiticide prescriptions and frequency of administration are tailored to the needs and risk level of the animal (4). These recommendations could also be applied to the countries studied herein.

### 4.1. Limitations

This study has several limitations that warrant discussion. Firstly, performing many more subgroup analysis (according to comorbidities, sex, age, etc.) would have been ideal in order to explain the high heterogeneity of our study. Secondly, many of the included studies have non-probabilistic sampling and small sample sizes, which could exaggerate the weighting representation in the random-effects method and alter the confidence interval values. Thirdly, future studies are needed to analyze more associated factors or compare different environmental (humidity, altitude, temperature, soil acidity), economic, and sociodemographic variables.

## 5. Conclusion

The current study found a high seroprevalence of human toxocariasis in Latin America and the Caribbean. Moreover, we observed differences in seroprevalence across Latin American and Caribbean countries probably attributed to sociodemographic and economic variables. Our findings can be used to establish epidemiological surveillance strategies for the prevention and early identification of this pathology. However, more factors associated with human toxocariasis seropositivity need to be analyzed in future studies.

## Author contributions

JU-B, AR-T, DC-P, PH-A, and VB-Z: conceptualization and writing review and editing. JU-B, EA-B, EH-B, XC-T, RS-A, and MH-C: data curation. JU-B, EA-B, and VB-Z: formal analysis. JU-B, EA-B, EH-B, and VB-Z: methodology. JU-B, EA-B, EH-B, XC-T, RS-A, MH-C, AR-T, DC-P, PH-A, and VB-Z: writing—original draft. All authors have read and agreed to the published version of the manuscript.

## Conflict of interest

The authors declare that the research was conducted in the absence of any commercial or financial relationships that could be construed as a potential conflict of interest.

## Publisher’s note

All claims expressed in this article are solely those of the authors and do not necessarily represent those of their affiliated organizations, or those of the publisher, the editors and the reviewers. Any product that may be evaluated in this article, or claim that may be made by its manufacturer, is not guaranteed or endorsed by the publisher.
