# Supplementary material for: Seroprevalence of human toxocariasis in Latin America and the Caribbean: a systematic review and meta-analysis
Source: Front Public Health. 2023 Jun 27;11:1181230. doi: 10.3389/fpubh.2023.1181230 (PMC10335805; doi:10.3389/fpubh.2023.1181230)
Supplement: Supplementary file 1 [file Table_1.DOCX]

Supplementary Material

Seroprevalence of human toxocariasis in Latin America and the Caribbean: A systematic review and meta-analysis

Juan R. Ulloque-Badaracco^1^, Enrique A. Hernandez-Bustamante^2,3^, Esteban A. Alarcón-Braga^1^, Miguel Huayta-Cortez^1^, Ximena L. Carballo-Tello^1^, Rosa A. Seminario-Amez^1^, Alejandra Rodríguez-Torres^4,5^, Donovan Casas-Patiño^4,5^, Percy Herrera-Añazco^6,7^, Vicente A. Benites-Zapata^8*^

*** Correspondence:** Vicente A Benites-Zapata vbenites@usil.edu.pe

**Table S1. Search Strategy**

| **Source** | **PubMed** |
| --- | --- |
| **Search** | **Formula** |
| **#1** | Toxocara canis [MH] OR toxocariasis [MH] OR human toxocariasis |
| **Source** | **Scopus** |
| **Search** | **Formula** |
| **#1** | TITLE-ABS-KEY (“Toxocara canis OR “toxocariasis OR “human toxocariasis) |
| **Source** | **Web of Science** |
| **Search** | **Formula** |
| **#1** | TI=(“ Toxocara canis OR toxocariasis OR AB=( human toxocariasis) |
| **Source** | **Embase** |
| **Search** | **Formula** |
| **#1** | 'Toxocara Canis'/exp OR (Toxocariasis OR human toxocariasis):ti |
| **Source** | **LILACS** |
| **Search** | **Formula** |
| **#1** | toxocara [Palavras] or toxocariasis [Palavras] and Human [Palavras] |
| **Source** | **Scielo** |
| **Search** | **Formula** |
| **#1** | (Toxocara) OR (Toxocariasis) AND (Human) |

**Table S2. Quality assessment of included studies**

|  | **NEWCASTLE - OTTAWA QUALITY ASSESSMENT SCALE FOR CROSS-SECTIONAL STUDIES** | | | | | | | | | | | |
| --- | --- | --- | --- | --- | --- | --- | --- | --- | --- | --- | --- | --- |
|  | | **SELECTION** | | | | **COMPARABILITY** | | **OUTCOME** | | |  |  |
| **STUDY** | | **Representativeness of the sample** | **Sample size** | **Non-respondents** | **Ascertainment of the exposure (risk factor)** | **The subjects in different outcome groups are comparable, based on the study design or analysis. Confounding factors are controlled.**  **Maximum : ☆☆** | | **Assessment of outcome** | | **Statistical test** | **SCORE** | **Evidence quality** |
| **Acero M et.al** | | ☆ | ☆ | ☆ | ☆ | ☆☆ | | ☆ | | ☆ | *8* | Low risk of bias |
| **Agudelo C et.al** | | ☆ | ☆ | ☆ | ☆ | ☆☆ | | ☆ | | ☆ | *8* | Low risk of bias |
| **Aguiar-Santos AM et.al** | | ☆ | ☆ | ☆ | ☆ | ☆☆ | | ☆ | | ☆ | *8* | Low risk of bias |
| **Alderete J et.al** | | ☆ | ☆ | ☆ | ☆ | ☆ | | ☆ | |  | *6* | High Risk of Bias |
| **Alonso JM et.al** | | ☆ | ☆ | ☆ | ☆ | ☆ | | ☆ | | ☆ | *7* | Low risk of bias |
| **Anaruma Filho F et.al** | | ☆ | ☆ | ☆ | ☆ | ☆ | ☆ | | ☆ | | *7* | Low risk of bias |
| **Araujo Z et.al** | | ☆ | ☆ | ☆ | ☆ | ☆ | ☆ | | ☆ | | *7* | Low risk of bias |
| **Archelli S et.al (A)** | | ☆ | ☆ | ☆ | ☆ | ☆ | ☆ | | ☆ | | *7* | Low risk of bias |
| **Archelli S et.al (B)** | | ☆ | ☆ | ☆ | ☆ | ☆ | ☆ | | ☆ | | *7* | Low risk of bias |
| **Baboolal S et.al** | | ☆ | ☆ | ☆ | ☆ | ☆ | ☆ | | ☆ | | *7* | Low risk of bias |
| **Berrocal J et.al** | | ☆ | ☆ | ☆ | ☆ | ☆ | ☆ | | ☆ | | *7* | Low risk of bias |
| **Bojanich MV et.al** | | ☆ | ☆ | ☆ | ☆ | ☆ | ☆ | | ☆ | | *7* | Low risk of bias |
| **Cabral M et.al** | | ☆ | ☆ | ☆ | ☆ | ☆☆ | ☆ | | ☆ | | *8* | Low risk of bias |
| **Campos-Junior D et.al** | | ☆ | ☆ | ☆ | ☆ | ☆☆ | ☆ | | ☆ | | *8* | Low risk of bias |
| **Cancrini G et.al** | | ☆ | ☆ | ☆ | ☆ | ☆☆ | ☆ | | ☆ | | *8* | Low risk of bias |
| **Cermeño J et.al** | | ☆ | ☆ | ☆ | ☆ | ☆ | ☆ | |  | | *6* | High Risk of Bias |
| **Chieffi P et.al** | | ☆ | ☆ | ☆ | ☆ | ☆ | ☆ | | ☆ | | *7* | Low risk of bias |
| **Chiodo P et.al** | | ☆ | ☆ | ☆ | ☆ | ☆☆ | ☆ | | ☆ | | *8* | Low risk of bias |
| **Coelho LM et.al.** | | ☆ | ☆ | ☆ | ☆ | ☆ | ☆ | | ☆ | | *7* | Low risk of bias |
| **Colli C et.al** | | ☆ | ☆ | ☆ | ☆ | ☆☆ | ☆ | | ☆ | | *8* | Low risk of bias |
| **Cook J et.al** | | ☆ | ☆ | ☆ | ☆ | ☆ | ☆ | | ☆ | | *7* | Low risk of bias |
| **Correa C et.al** | | ☆ | ☆ | ☆ | ☆ | ☆☆ | ☆ | | ☆ | | *8* | Low risk of bias |
| **Damian M et.al** | | ☆ | ☆ | ☆ | ☆ | ☆☆ | ☆ | | ☆ | | *8* | Low risk of bias |
| **Dattoli V et.al** | | ☆ | ☆ | ☆ | ☆ | ☆ | ☆ | | ☆ | | *7* | Low risk of bias |
| **De Abreu A et.al** | | ☆ | ☆ | ☆ | ☆ | ☆☆ | ☆ | | ☆ | | *8* | Low risk of bias |
| **Devera R et.al** | | ☆ | ☆ | ☆ | ☆ | ☆☆ | ☆ | | ☆ | | *8* | Low risk of bias |
| **Díaz-Suárez O et.al** | | ☆ | ☆ | ☆ | ☆ | ☆☆ | ☆ | | ☆ | | *8* | Low risk of bias |
| **Espinoza Y et.al (B)** | | ☆ | ☆ | ☆ | ☆ | ☆ | ☆ | | ☆ | | *7* | Low risk of bias |
| **Espinoza Y et.al (A)** | | ☆ | ☆ | ☆ | ☆ | ☆ | ☆ | | ☆ | | *7* | Low risk of bias |
| **Espinoza Y et.al (C)** | | ☆ | ☆ | ☆ | ☆ | ☆☆ | ☆ | | ☆ | | *8* | Low risk of bias |
| **Figuereido S et.al** | | ☆ | ☆ | ☆ | ☆ | ☆☆ | ☆ | | ☆ | | *8* | Low risk of bias |
| **Fillaux J et.al** | | ☆ | ☆ | ☆ | ☆ | ☆☆ | ☆ | | ☆ | | *8* | Low risk of bias |
| **García-Pedrique ME et.al** | | ☆ | ☆ | ☆ | ☆ | ☆☆ | ☆ | | ☆ | | *8* | Low risk of bias |
| **Gétaz-Schaller L et.al** | | ☆ | ☆ | ☆ | ☆ | ☆ | ☆ | | ☆ | | *7* | Low risk of bias |
| **Guilherme E et.al** | | ☆ | ☆ | ☆ | ☆ | ☆☆ | ☆ | | ☆ | | *8* | Low risk of bias |
| **Guo F et.al** | | ☆ | ☆ | ☆ | ☆ | ☆☆ | ☆ | | ☆ | | *8* | Low risk of bias |
| **Heredia R et.al** | | ☆ | ☆ | ☆ | ☆ | ☆☆ | ☆ | | ☆ | | *8* | Low risk of bias |
| **Hernández S et.al** | | ☆ | ☆ | ☆ | ☆ | ☆ | ☆ | | ☆ | | *7* | Low risk of bias |
| **Kanobana K et.al** | | ☆ | ☆ | ☆ | ☆ | ☆☆ | ☆ | | ☆ | | *8* | Low risk of bias |
| **Lescano S et.al** | | ☆ | ☆ | ☆ | ☆ | ☆☆ | ☆ | | ☆ | | *8* | Low risk of bias |
| **Lima-Coêlho R et.al** | | ☆ | ☆ | ☆ | ☆ | ☆☆ | ☆ | | ☆ | | *8* | Low risk of bias |
| **Lopez MA et.al** | | ☆ | ☆ | ☆ | ☆ | ☆☆ | ☆ | | ☆ | | *8* | Low risk of bias |
| **Lozano-Beltrán D et.al** | | ☆ | ☆ | ☆ | ☆ | ☆ | ☆ | | ☆ | | *7* | Low risk of bias |
| **Lynch NR et.al (A)** | | ☆ | ☆ | ☆ | ☆ | ☆ | ☆ | | ☆ | | *7* | Low risk of bias |
| **Lynch NR et.al (B)** | | ☆ | ☆ | ☆ | ☆ | ☆☆ | ☆ | | ☆ | | *8* | Low risk of bias |
| **Manini M et.al** | | ☆ | ☆ | ☆ | ☆ | ☆☆ | ☆ | | ☆ | | *8* | Low risk of bias |
| **Marchioro A et.al** | | ☆ | ☆ | ☆ | ☆ | ☆ | ☆ | | ☆ | | *7* | Low risk of bias |
| **Martín U et.al (A)** | | ☆ | ☆ | ☆ | ☆ | ☆☆ | ☆ | | ☆ | | *8* | Low risk of bias |
| **Martín U et.al (B)** | | ☆ | ☆ | ☆ | ☆ | ☆☆ | ☆ | | ☆ | | *8* | Low risk of bias |
| **Martínez M et.al** | | ☆ | ☆ | ☆ | ☆ | ☆ | ☆ | | ☆ | | *7* | Low risk of bias |
| **Mattia S et.al** | | ☆ | ☆ | ☆ | ☆ | ☆☆ | ☆ | | ☆ | | *8* | Low risk of bias |
| **Meza D et.al** | | ☆ | ☆ | ☆ | ☆ | ☆☆ | ☆ | | ☆ | | *8* | Low risk of bias |
| **Minvielle M et.al** | | ☆ | ☆ | ☆ | ☆ | ☆ | ☆ | | ☆ | | *7* | Low risk of bias |
| **Miranda-Choque E et.al** | | ☆ | ☆ | ☆ | ☆ | ☆ | ☆ | | ☆ | | *7* | Low risk of bias |
| **Montalvo A et.al** | | ☆ | ☆ | ☆ | ☆ | ☆☆ | ☆ | | ☆ | | *8* | Low risk of bias |
| **Morocoima A et.al** | | ☆ | ☆ | ☆ | ☆ | ☆☆ | ☆ | | ☆ | | *8* | Low risk of bias |
| **Muñoz-Guzmán M et.al** | | ☆ | ☆ | ☆ | ☆ | ☆ | ☆ | | ☆ | | *7* | Low risk of bias |
| **Muradian V et.al** | | ☆ | ☆ | ☆ | ☆ | ☆☆ | ☆ | | ☆ | | *8* | Low risk of bias |
| **Nava-Cortés N et.al** | | ☆ | ☆ | ☆ | ☆ | ☆ | ☆ | | ☆ | | *7* | Low risk of bias |
| **Negri E et.al** | | ☆ | ☆ | ☆ | ☆ | ☆☆ | ☆ | | ☆ | | *8* | Low risk of bias |
| **Oliart-Guzmán H et.al** | | ☆ | ☆ | ☆ | ☆ | ☆☆ | ☆ | | ☆ | | *8* | Low risk of bias |
| **Orlando-Indacochea N et.al** | | ☆ | ☆ | ☆ | ☆ | ☆ | ☆ | |  | | *6* | High Risk of Bias |
| **Ortega-Pacheco A et.al** | | ☆ | ☆ | ☆ | ☆ | ☆☆ | ☆ | | ☆ | | *8* | Low risk of bias |
| **Oviedo-Vera A et.al** | | ☆ | ☆ | ☆ | ☆ | ☆☆ | ☆ | | ☆ | | *8* | Low risk of bias |
| **Paludo M et.al** | | ☆ | ☆ | ☆ | ☆ | ☆ | ☆ | | ☆ | | *7* | Low risk of bias |
| **Paranhos-Fragoso et.al** | | ☆ | ☆ | ☆ | ☆ | ☆ | ☆ | | ☆ | | *7* | Low risk of bias |
| **Pereira L et.al** | | ☆ | ☆ | ☆ | ☆ | ☆☆ | ☆ | | ☆ | | *8* | Low risk of bias |
| **Prestes-Carneiro L et.al (A)** | | ☆ | ☆ | ☆ | ☆ | ☆☆ | ☆ | | ☆ | | *8* | Low risk of bias |
| **Prestes-Carneiro L et.al (B)** | | ☆ | ☆ | ☆ | ☆ | ☆☆ | ☆ | | ☆ | | *8* | Low risk of bias |
| **Prestes-Carneiro L et.al (C)** | | ☆ | ☆ | ☆ | ☆ | ☆ | ☆ | | ☆ | | *7* | Low risk of bias |
| **Pulcha-Ugarte R et.al** | | ☆ | ☆ | ☆ | ☆ | ☆ | ☆ | | ☆ | | *7* | Low risk of bias |
| **Radman NE et.al** | | ☆ | ☆ | ☆ | ☆ | ☆☆ | ☆ | | ☆ | | *8* | Low risk of bias |
| **Ramírez-Bustamante C et.al** | | ☆ | ☆ | ☆ | ☆ | ☆☆ | ☆ | | ☆ | | *8* | Low risk of bias |
| **Ribeiro L et.al** | | ☆ | ☆ | ☆ | ☆ | ☆☆ | ☆ | | ☆ | | *8* | Low risk of bias |
| **Rivarola M et.al** | | ☆ | ☆ | ☆ | ☆ | ☆ | ☆ | |  | | *6* | High Risk of Bias |
| **Rodriguez C et.al** | | ☆ | ☆ | ☆ | ☆ | ☆☆ | ☆ | | ☆ | | *8* | Low risk of bias |
| **Roldan W et.al (A)** | | ☆ | ☆ | ☆ | ☆ | ☆☆ | ☆ | | ☆ | | *8* | Low risk of bias |
| **Roldan W et.al (B)** | | ☆ | ☆ | ☆ | ☆ | ☆ | ☆ | | ☆ | | *7* | Low risk of bias |
| **Roldan W et.al (C)** | | ☆ | ☆ | ☆ | ☆ | ☆☆ | ☆ | | ☆ | | *8* | Low risk of bias |
| **Romero R et.al** | | ☆ | ☆ | ☆ | ☆ | ☆ | ☆ | | ☆ | | *7* | Low risk of bias |
| **Romero-Nuñez C et.al** | | ☆ | ☆ | ☆ | ☆ | ☆☆ | ☆ | | ☆ | | *8* | Low risk of bias |
| **Rubinsky-Elefant G et.al** | | ☆ | ☆ | ☆ | ☆ | ☆ | ☆ | | ☆ | | *7* | Low risk of bias |
| **Santarem V et.al** | | ☆ | ☆ | ☆ | ☆ | ☆☆ | ☆ | | ☆ | | *8* | Low risk of bias |
| **Santos P et.al** | | ☆ | ☆ | ☆ | ☆ | ☆ | ☆ | | ☆ | | *7* | Low risk of bias |
| **Sariego I et.al** | | ☆ | ☆ | ☆ | ☆ | ☆☆ | ☆ | | ☆ | | *8* | Low risk of bias |
| **Schoenardie E et.al** | | ☆ | ☆ | ☆ | ☆ | ☆ | ☆ | | ☆ | | *7* | Low risk of bias |
| **Souza RF et.al** | | ☆ | ☆ | ☆ | ☆ | ☆☆ | ☆ | | ☆ | | *8* | Low risk of bias |
| **Taranto NJ et.al (A)** | | ☆ | ☆ | ☆ | ☆ | ☆☆ | ☆ | | ☆ | | *8* | Low risk of bias |
| **Taranto NJ et.al (B)** | | ☆ | ☆ | ☆ | ☆ | ☆☆ | ☆ | | ☆ | | *8* | Low risk of bias |
| **Thompson DE et.al** | | ☆ | ☆ | ☆ | ☆ | ☆☆ | ☆ | | ☆ | | *8* | Low risk of bias |
| **Tinoco-Gracias L et.al** | | ☆ | ☆ | ☆ | ☆ | ☆ | ☆ | | ☆ | | *7* | Low risk of bias |
| **Triviño X et.al** | | ☆ | ☆ | ☆ | ☆ | ☆ | ☆ | |  | | *6* | High Risk of Bias |
| **Urbano-Ferreira M et.al** | | ☆ | ☆ | ☆ | ☆ | ☆ | ☆ | | ☆ | | *7* | Low risk of bias |
| **Vargas C et.al** | | ☆ | ☆ | ☆ | ☆ | ☆☆ | ☆ | | ☆ | | *8* | Low risk of bias |
| **Virginia P et.al** | | ☆ | ☆ | ☆ | ☆ | ☆ | ☆ | | ☆ | | *7* | Low risk of bias |
| **Waindok P et.al** | | ☆ | ☆ | ☆ | ☆ | ☆☆ | ☆ | | ☆ | | *8* | Low risk of bias |

|  | |  | | **NEWCASTLE - OTTAWA QUALITY ASSESSMENT SCALE FOR CASE-CONTROL STUDIES** | | | | | | | | | |
| --- | --- | --- | --- | --- | --- | --- | --- | --- | --- | --- | --- | --- | --- |
|  | | **SELECTION** | | | | **COMPARABILITY** | | **EXPOSURE** | |  |  |  |  |
| **STUDY** | | **Is the case definition adequate?** | | **Representativeness of the cases** | **Selection of Controls** | **Definition of Controls** | **Comparability of cases and controls on the basis of the design or analysis (Maximum : ☆☆ )** |  | **Same method of ascertainment for cases and controls** | **Non-Response rate** | **Ascertainment of exposure** | **SCORE** | **Evidence quality** |
| **Alvarado-Esquivel C et.al (A)** | | ☆ | | ☆ | ☆ | ☆ | ☆ | | ☆ | ☆ | ☆ | 8 | Low risk of bias |
| **Alvarado-Esquivel C et.al (B)** | | ☆ | | ☆ | ☆ | ☆ | ☆ | | ☆ | ☆ | ☆ | 8 | Low risk of bias |
| **Alvarado-Esquivel C et.al (C)** | | ☆ | | ☆ | ☆ | ☆ | ☆ | | ☆ | ☆ | ☆ | 8 | Low risk of bias |
| **Silva-Cadore P et.al** | | ☆ | | ☆ | ☆ | ☆ | ☆ | | ☆ | ☆ | ☆ | 8 | Low risk of bias |
| **Nicoletti A et.al** | | ☆ | | ☆ | ☆ | ☆ | ☆ | | ☆ | ☆ | ☆ | 8 | Low risk of bias |
